# Supplementary material for: Algebraic methods and computational strategies for pseudoinverse-based MR image reconstruction (Pinv-Recon)
Source: Sci Rep. 2025 Oct 30;15:37997. doi: 10.1038/s41598-025-21929-z (PMC12575614; doi:10.1038/s41598-025-21929-z)
Supplement: Supplementary file 1 — Supplementary Information. [file 41598_2025_21929_MOESM1_ESM.pdf]

Supplementary Materials

1 Versatility of Pinv-Recon for Different K-Space Trajectories

Figure S1 demonstrates the versatility of Pinv-Recon for different k-space sampling trajectories, plotting from top to bottom the trajectory, its SVD spectrum, the reconstructed image, the SRF maps, and the Noise matrices. The SVD spectra show that Cartesian sampling is the best-conditioned, as seen also in its artifact-free reconstruction of the Shepp-Logan phantom. Pinv-Recon also allowed the direct calculation of the SRFs of each trajectory, showing all pixels are fully represented in the reconstructed image. The noise matrices reflect that noise is amplified in the edges of the reconstructed image for the radial and spiral trajectories.

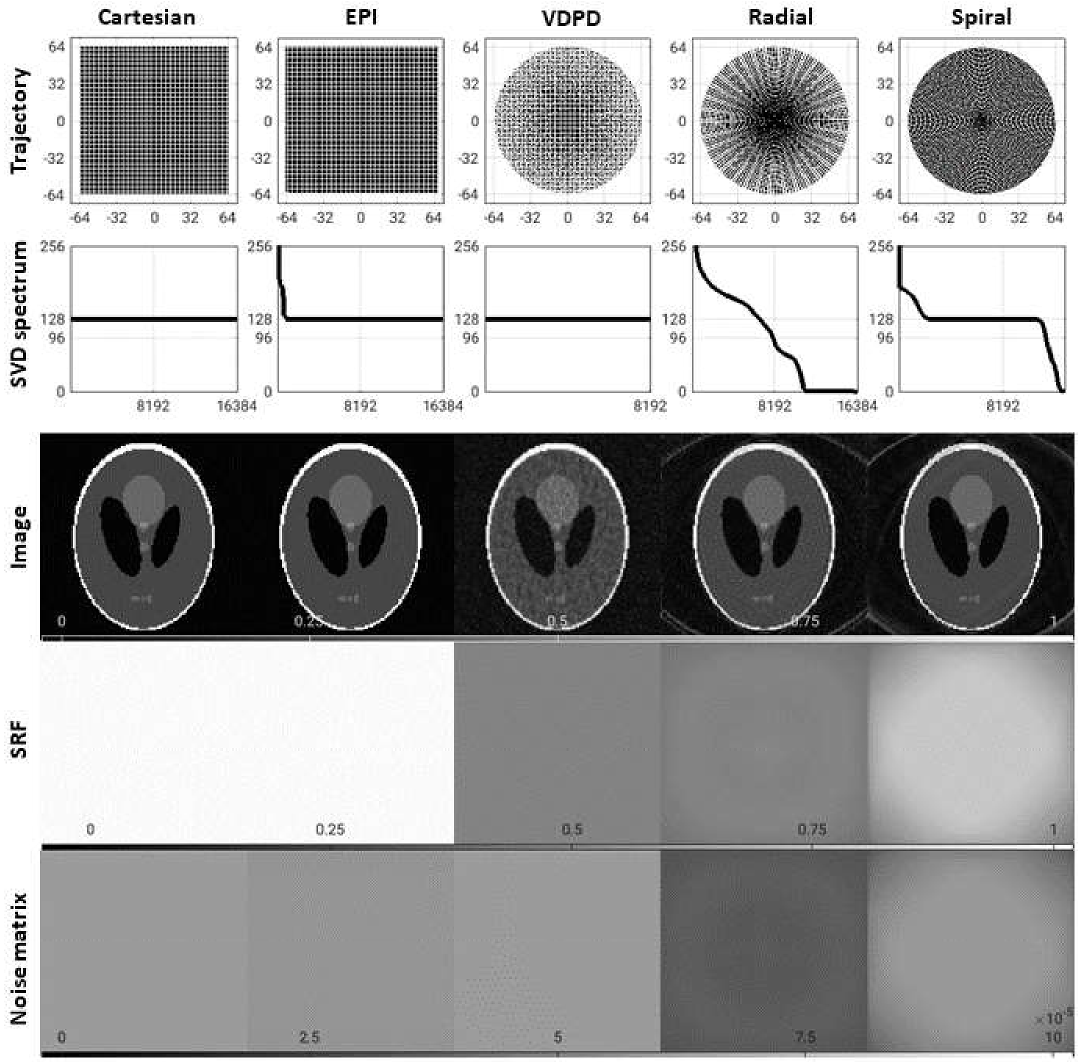

Figure S1. Demonstration of Pinv-Recon on a variety of k-space sampling trajectories. From left to right: Cartesian, EPI, Variable-Density Poisson Disk (VDPD), radial, spiral. From top to bottom: sampling trajectory, SVD, reconstructed Shepp-Logan image, SRF, noise matrix.

2 Computational Evaluation on a Mobile Workstation

Section 3.1 listed the computational times required for a high-performance workstation similar to the reconstruction machines found on MRI scanners. Here, we show that even modern mobile workstations with lower-performance can manage the direct pseudoinversion of various encoding matrices. We perform the same computational evaluation described in section 3.1.1 on a Precision 7680 mobile workstation(Dell, Texas, USA), which has 13th Gen Intel®Core^{TM} i9-13950HX, 2200 Mhz, 24 Core(s), 32 Logical Processor(s), 64.0 GB of Installed Physical Memory (RAM), and an NVIDIA®RTX^{TM} 2000 Ada Generation Laptop GPU with 16GB.

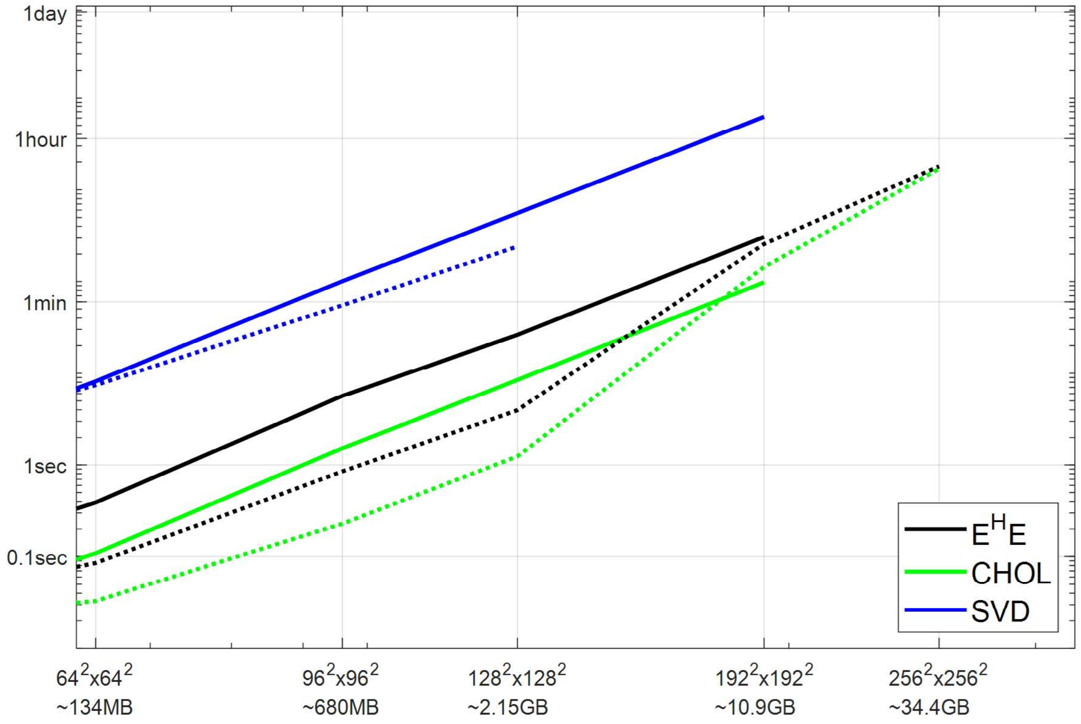

N_k x N_r	64^2 x 64^2		96^2 x 96^2		128^2 x 128^2		192^2 x 192^2		256^2 x 256^2	
**Memory [GB]** (single precision)	0.13		0.67		2.14		10.87		34.35	
**Equivalent MTX** (1D/2D/3D)	4096 / 64^2 / 16^3		9216 / 96^2 / 21^3		16384 / 128^2 / 25^3		36864 / 192^2 / 33^3		65536 / 256^2 / 40^3	
	CPU	GPU	CPU	GPU	CPU	GPU	CPU	GPU	CPU	GPU
**SVD**	0.164	0.416	8.184	7.438	100.813	55.011	557.370	241.993	6199.771	
**QR**	0.082	0.257	3.520	2.408	42.806	17.893	249.796	169.173	2718.892	
**EHE**	0.012	0.010	0.390	0.085	5.672	0.852	26.173	3.963	308.141	
**Eig**	0.057	0.085	2.521	1.509	34.304	11.809	201.932	55.214	2175.329	
**Chol**	0.003	0.012	0.109	0.033	1.524	0.229	8.461	1.243	98.099	

Figure S2. Graph showing computational times for matrix decomposition on a mobile workstation

3 B_0 Correction Examples

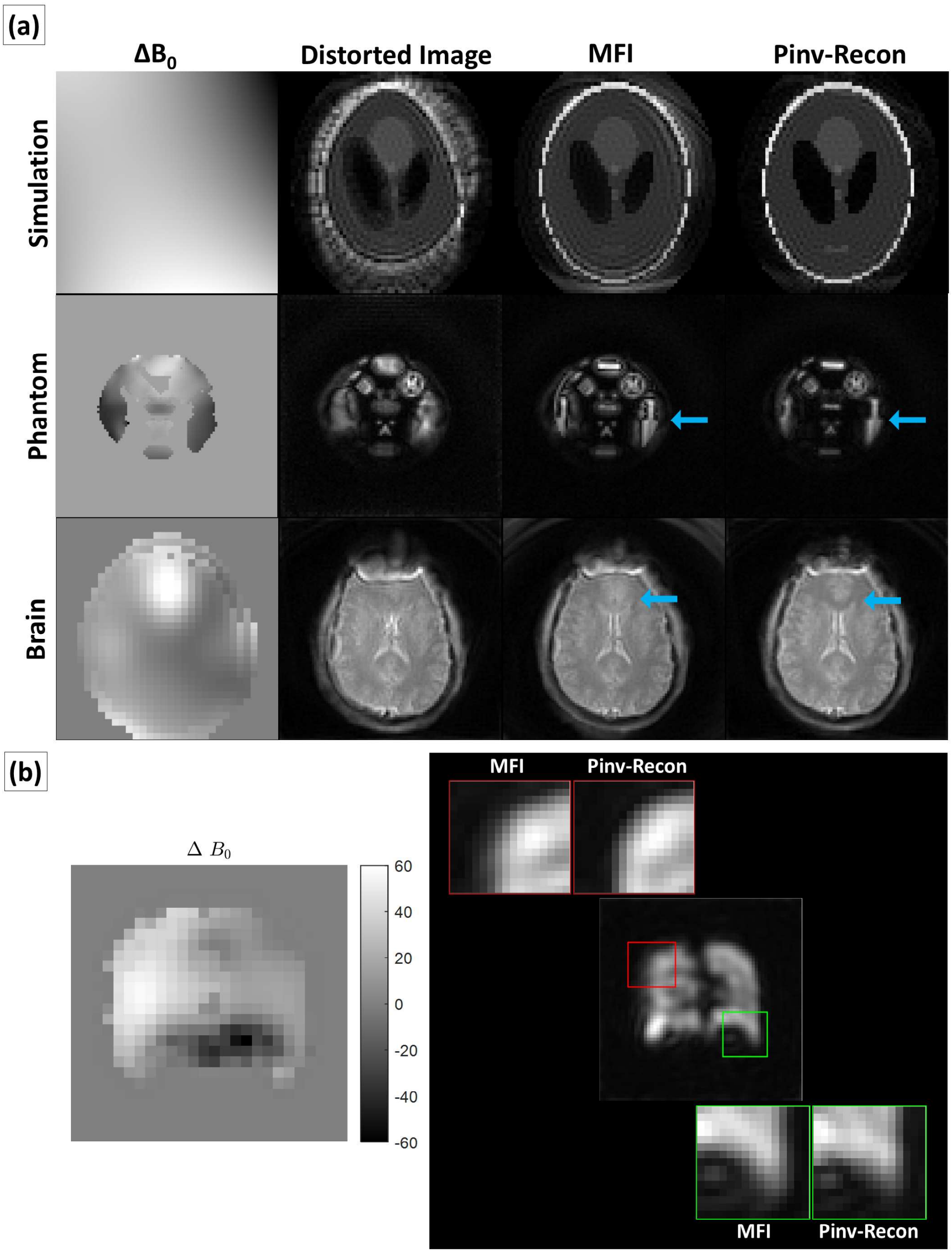

Figure S3. (a) Shepp-Logan simulations, Structural Phantom acquisitions, and in vivo brain acquisitions comparing correction of B_0 distortion using Pinv-Recon versus MFI (b) Left: Off-Resonance Map. Right: Low-resolution hyperpolarized Xenon-129 image, with the original image shown in the center and the B_0 corrected images using MFI and Pinv-Recon shown outside

	MTX 48		MTX 64		MTX 96	
	**Pinv-Recon**	**Gridding**	**Pinv-Recon**	**Gridding**	**Pinv-Recon**	**Gridding**
**MSE**	0.001343	0.001667	0.000971	0.001736	0.000877	0.002350
**PSNR**	28.72	27.78	30.12	27.60	30.57	26.29
**SSIM**	0.8194	0.7859	0.8206	0.7815	0.8465	0.7670

Table 1. Results comparing B_0 correction using Pinv-Recon versus using gridding with MFI correction in a structured resolution phantom.

Figure S3 shows two examples of including B_0 correction in the Pinv-Recon encoding matrix, compared to B_0 with Multifrequency Interpolation (MFI)[6]. Figure S3a shows B_0 correction in a Shepp-Logan simulation, a structured resolution phantom and in vivo a human brain scan at the proton frequency. The off-resonance maps are shown in column 1, the distorted images are shown in column 2. By incorporating the B_0 maps into Pinv-Recon and into an MFI correction for the image obtained through gridding reconstruction, the blurring effects can be ameliorated, recovering images close to the reference image. Pinv-Recon results in lower MSE, higher PSNR, and higher SSIM than MFI for all of the matrix sizes (Table 1).

Figure S3b shows low resolution 2D spiral hyperpolarized Xenon-129 lung dataset, which had a longer readout time. Off-resonance blurring can be observed in the original image. Using gridding reconstruction with MFI correction and using Pinv-Recon with embedded B_0 encoding improved image quality, but Pinv-Recon sharpens the image to a greater extent.

The acquisition details for the proton dataset were maximum gradient amplitude = 30 mT/m; slew rate = 120 T/m/s; FOV = 240×240 mm; bandwidth = 250 kHz. Acquisition parameters: Flip Angle = 30°; TR = minimum TR; Slice thickness = 3mm). The matrix 48 four-arm spiral was repeated at five different TEs ([0,1,2,5,10]ms) to iteratively fit for a B_0 map using the MEDI toolbox. The phantom was imaged in a 3T GE Premier scanner and using a 5-channel flexible AIR coil (GE HealthCare, WI). The healthy volunteer (Male; Age 30) was scanned in the same scanner using a 16-channel receive-only headcoil (GE HealthCare, WI), using the same single-arm MTX96 spiral and the four-arm MTX 48 spiral.

Those for the low-resolution 2D spiral integrated into the Transmit Gain Calibration of a hyperpolarized Xenon-129 scan, which used a 10% dose of Xenon. The participant (Female; Age 24) inhaled 1 L hyperpolarized gas containing a mixture of xenon, polarized for ∼10 min, and nitrogen (0.1:0.9 L, respectively). FOV = 400×400 mm, 1.8 ms partially self-refocused excitation pulse, TR = 230 ms, bandwidth = 250 kHz. The B_0 map was determined by repeating this acquisition at echo times of 1.4, 2.3, 3.9, 6.5, 10.8, 18, 30, 50ms then fitting with the MEDI toolbox.

4 Arterial Spin Labelling Example

Other suitable applications for Pinv-Recon are medium-resolution functional imaging applications such as Arterial Spin Labelling (ASL). This figure shows an example of reconstructing a stack-of-spirals ASL dataset (MTX $128^2 \times 42$, FOV = 240 $\times$ 240 mm, 8 arms, TR = 4.8 s, TE = 10 ms).

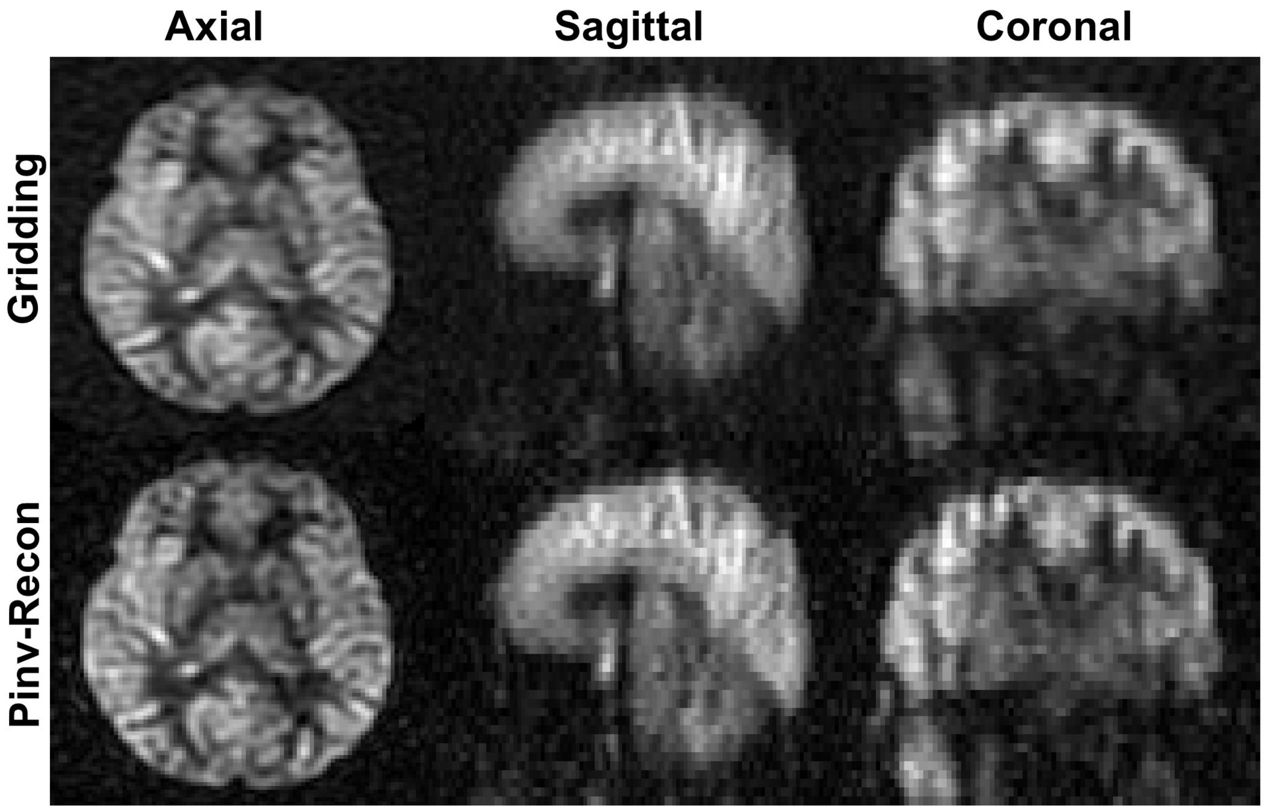

Figure S4. Example of reconstructing ASL data using Griding Pinv-Recon.
